# Study on a kind of *ϕ*-Laplacian Liénard equation with attractive and repulsive singularities

**DOI:** 10.1186/s13660-017-1410-3

**Published:** 2017-08-03

**Authors:** Yun Xin, Zhibo Cheng

**Affiliations:** 1 College of Computer Science and Technology, Henan Polytechnic University, Jiaozuo, China; 2 School of Mathematics and Information Science, Henan Polytechnic University, Jiaozuo, 454000 China; 3 Department of Mathematics, Sichuan University, Chengdu, 610064 China

**Keywords:** 34K14, 34C25, positive periodic solution, *ϕ*-Laplacian, attractive singularity, repulsive singularity, Liénard equation

## Abstract

In this paper, by application of the Manasevich-Mawhin continuation theorem, we investigate the existence of a positive periodic solution for a kind of *ϕ*-Laplacian singular Liénard equation with attractive and repulsive singularities.

## Introduction

Liénard equation [1]

1.1 $$ { } x'' + f(x)x' + g(x) = 0 $$

appears as a simplified model in many domains in science and engineering. It was intensively studied during the first half of the 20th century as it can be used to model oscillating circuits or simple pendulums. For example, Van der Pol oscillator

$$x''-\mu\bigl(1-x^{2}\bigr)x'+x=0 $$

is a Liénard equation.

From then on, there have been a good amount of work on periodic solutions for Liénard equations (see [2–12] and the references cited therein). Some classical tools have been used to study Liénard equation in the literature, including Mawhin’s coincidence degree theorem [2, 3, 5], topological degree methods [4, 6], Schauder’s fixed point theorem [7], Massera’s theorem [8], the Manasevich-Mawhin continuation theorem [9, 12], generalized polar coordinates [10] and the Poincaré map [11].

At the same time, some authors began to consider Liénard equation with singularity [13–20]. For example, in 1996, Zhang [20]  discussed a kind of singular Liénard equation

1.2 $$ { } x''+f(x)x'+g(t,x)=0, $$

where *g* was on the singular case, i.e., when $g(t,x)\rightarrow+\infty$, as $x\rightarrow0^{+}$. By application of coincidence degree theory, the author obtained that (1.2) had at least one periodic solution. Afterwards, Jebelean and Mawhin investigated the following quasilinear equation of *p*-Laplacian type:

$$\bigl( \bigl\vert x' \bigr\vert ^{p-2}x' \bigr)'+f(x)x'(t)+g(x)=h(t), $$

where *g* satisfied slightly strong singularity, i.e.,

$$\int^{1}_{0}g(u)\,du=-\infty. $$

The authors proved that the above problem had at least one positive periodic solution through a basic application of the Manasevich-Mawhin continuation theorem. Recently, Xin and Cheng [19] studied the following *p*-Laplacian Liénard equation with singularity and deviating argument:

1.3 $$ { } \bigl( \bigl\vert x' \bigr\vert ^{p-2}x'\bigr)'+f(x)x'+g \bigl(t,x(t-\sigma)\bigr)=e(t). $$

By applications of coincidence degree theory and some analysis skills, they obtained that (1.3) had at least one positive periodic solution.

All the aforementioned results concern singular Liénard equation and singular *p*-Laplacian Liénard equation. There are few results on the *ϕ*-Laplacian Liénard equation with singularity. Motivated by [13, 19, 20], in this paper, we further consider the following *ϕ*-Laplacian Liénard equation:

1.4 $$ { } \bigl(\phi\bigl(x'(t)\bigr)\bigr)'+f \bigl(t,x(t)\bigr)x'(t)+g\bigl(x(t-\sigma)\bigr)=e(t), $$

where $f:\mathbb{R}\to\mathbb{R}$ is an $L^{2}$-Carathéodory function, which means, it is measurable in the first variable and continuous in the second variable, and for every $0< r< s$, there exists $h_{r,s}\in L^{2}[0,T]$ such that $\vert g(t,x(t)) \vert \leq h_{r,s}$ for all $x\in[r,s]$ and a.e. $t\in[0,T]$; *τ* is a positive constant; $e\in L^{2}(\mathbb{R})$ is a *T*-periodic function; $g:(0,+\infty)\to \mathbb{R}$ is the $L^{2}$-function, the nonlinear term *g* of (1.4) can be with a singularity at origin, i.e.,

$$ \lim_{x\rightarrow0^{+}} g(x)=+\infty, \quad \Bigl(\mbox{or } \lim _{x\rightarrow0^{+}} g(x)=-\infty\Bigr), \quad \mbox{uniformly in } t. $$

It is said that (1.4) is of attractive type (resp. repulsive type) if $g(x)\rightarrow+\infty$ (resp. $g(x)\rightarrow-\infty$) as $x\rightarrow0^{+}$.

Moreover, $\phi:\mathbb{R}\rightarrow\mathbb{R}$ is a continuous function and $\phi(0)=0$, which satisfies

$(A_{1})$
$(\phi(x_{1})-\phi(x_{2}))(x_{1}-x_{2})>0$ for $\forall x_{1}\neq x_{2}, x_{1}, x_{2}\in\mathbb{R}$;
$(A_{2})$
There exists a function $\alpha:[0,+\infty]\rightarrow[0,+\infty], \alpha(s)\rightarrow+\infty$ as $s\rightarrow+\infty$, such that $\phi(x)\cdot x\geq\alpha( \vert x \vert ) \vert x \vert $ for $\forall x\in\mathbb{R}$.

It is easy to see that *ϕ* represents a large class of nonlinear operators, including $\vert u \vert ^{p-2}u: \mathbb{R}\rightarrow\mathbb{R} $ which is a *p*-Laplacian operator.

The remaining part of the paper is organized as follows. In Section 2, we give some preliminary lemmas. In Section 3, by employing the Manasevich-Mawhin continuation theorem, we state and prove the existence of a positive periodic solution for (1.4) with attractive singularity. In Section 4, we investigate the existence result for (1.4) with repulsive singularity. In Section 5, two numerical examples demonstrate the validity of the method. Our results improve and extend the results in [13, 15, 18–20].

## Preliminary lemmas

For the *T*-periodic boundary value problem

2.1 $$ { } \bigl(\phi\bigl(x'(t)\bigr)\bigr)'= \tilde{f}\bigl(t,x,x'\bigr), $$

here $\tilde{f}:[0,T]\times\mathbb{R}\times\mathbb{R}\rightarrow\mathbb{R}$ is assumed to be Carathéodory.

### Lemma 2.1

Manasevich-Mawhin [21]

*Let* Ω *be an open bounded set in* $C^{1}_{T}:=\{x\in C^{1}(\mathbb{R},\mathbb{R}): x\textit{ is $T$-periodic}\}$. *If*

(i) *for each* $\lambda\in(0,1)$, *the problem*
  $$\bigl(\phi\bigl(x'\bigr)\bigr)'=\lambda \tilde{f} \bigl(t,x,x'\bigr), \qquad x(0)=x(T), \qquad x'(0)=x'(T) $$
  *has no solution on* *∂*Ω;
(ii) *the equation*
  $$F(a):=\frac{1}{T} \int^{T}_{0}\tilde{f}\bigl(t,x,x' \bigr)\,dt=0 $$
  *has no solution on* $\partial\Omega\cap\mathbb{R}$;
(iii) *the Brouwer degree of F*
  $$\deg\{F,\Omega\cap\mathbb{R},0\}\neq0, $$
  *then the periodic boundary value problem* (2.1) *has at least one periodic solution on* Ω̄.

Next, we embed equation (1.4) into the following equation family with a parameter $\lambda\in(0,1]$:

2.2 $$ { } \bigl(\phi\bigl(x'(t)\bigr)\bigr)'+ \lambda f\bigl(t,x(t)\bigr)x'(t)+\lambda g\bigl(x(t-\tau)\bigr)= \lambda e(t). $$

By applications of Lemma 2.1, we obtain the following result.

### Lemma 2.2

*Suppose that* $(A_{1})$ *and* $(A_{2})$ *hold*. *Assume that there exist positive constants* $E_{1}, E_{2}, E_{3}$ *and* $E_{1}< E_{2}$ *such that the following conditions hold*:

1. *Each possible periodic solution* *x* *to equation* (2.2) *such that* $E_{1}< x(t)< E_{2}$, *for all* $t\in[0,T]$ *and* $\Vert x' \Vert < E_{3}$, *here* $\Vert x' \Vert :=\max_{t\in[0,T]} \vert x'(t) \vert $.
2. *Each possible solution* *C* *to equation*
  $$g(C)-\frac{1}{T} \int^{T}_{0} e(t)\,dt=0 $$
  *satisfies* $E_{1}< C< E_{2}$.
3. *It holds*
  $$\biggl(g(E_{1})-\frac{1}{T} \int^{T}_{0} e(t)\,dt \biggr) \biggl(g(E_{2})- \frac {1}{T} \int^{T}_{0} e(t)\,dt \biggr)< 0. $$
  *Then* (1.4) *has at least one* *T*-*periodic solution*.

## Main results (I): periodic solution of (1.4) with attractive singularity

In this section, we investigate the existence of a positive periodic solution for (1.4) with attractive singularity.

### Theorem 3.1

*Assume that conditions* $(A_{1})$ *and* $(A_{2})$ *hold*. *Suppose that the following conditions hold*:

$(H_{1})$
*There exist constants* $0< d_{1}< d_{2}$ *such that* $g(x)-e(t)>0$ *for* $x\in(0,d_{1})$ *and* $g(x)-e(t)<0$ *for* $x\in(d_{2},+\infty)$.
$(H_{2})$
*There exist positive constants* $a, b$ *and* *m* *such that*
  3.1 $$ g(x)\leq a x^{m}+b, \quad \textit{for all } x>0. $$
$(H_{3})$
(*Attractive singularity*) $\lim_{x\to 0^{+}}\int^{1}_{x}g(s)\,ds=+\infty$.
$(H_{4})$
*There exists a constant* $\gamma>0$ *such that* $\inf_{x\in\mathbb{R}} \vert f(t,x) \vert \geq\gamma>0$.
*Then* (1.4) *has a positive* *T*-*periodic solution*.

### Proof

Firstly, we claim that there exists a point $t_{1}\in[0,T]$ such that

3.2 $$ { } d_{1}\leq x(t_{1})\leq d_{2}. $$

Let $\underline{t}$, *t̅* be, respectively, the global minimum point and the global maximum point $x(t)$ on $[0,T]$; then $x'(\underline{t})=0$ and $x'(\overline{t})=0$, and we claim that

3.3 $$ { } \bigl(\phi\bigl(x'(\underline{t})\bigr) \bigr)'\geq0. $$

In fact, if (3.3) does not hold, then $(\phi(x'(\underline{t})))'<0$ and there exists $\varepsilon>0$ such that $(\phi(x'(t)))'<0$ for $t\in(\underline{t}-\varepsilon,\underline{t}+\varepsilon)$. Therefore $\phi(x'(t))$ is strictly decreasing for $t\in(\underline{t}-\varepsilon,\underline{t}+\varepsilon)$. From $(A_{1})$, we know that $x'(t)$ is strictly decreasing for $t\in(\underline{t}-\varepsilon,\underline{t}+\varepsilon)$. This contradicts the definition of $\underline{t}$. Thus, (3.3) is true. From (2.2) and (3.3), we have

3.4 $$ { } g\bigl(x(\underline{t}-\tau)\bigr)-e(\underline{t})\leq0. $$

Similarly, we can get

3.5 $$ { } g\bigl(x(\overline{t}-\tau)\bigr)-e(\overline{t})\geq0. $$

From $(H_{1})$, (3.4) and (3.5), we have

$$x(\underline{t}-\tau)\geq d_{1}\quad \mbox{and}\quad x(\overline{t}-\tau)\leq d_{2}. $$

In view of *x* being a continuous function, we can get (3.2).

Multiplying both sides of (2.2) by $x'(t)$ and integrating over the interval $[0,T]$, we have

3.6 $$\begin{aligned} { } &\int^{T}_{0}\bigl(\phi\bigl(x'(t)\bigr) \bigr)'x'(t)\,dt+\lambda \int ^{T}_{0}f\bigl(t,x(t)\bigr) \bigl\vert x'(t) \bigr\vert ^{2}\,dt+\lambda \int^{T}_{0}g\bigl(x(t-\tau)\bigr)x'(t)\,dt \\ &\quad= \lambda \int ^{T}_{0}e(t)x'(t)\,dt. \end{aligned}$$

Moreover, we have

3.7 $$\begin{aligned} { } \int^{T}_{0}\bigl(\phi\bigl(x'(t)\bigr) \bigr)'x'(t)\,dt&= \int^{T}_{0}x'(t)\,d\bigl(\phi \bigl(x'(t)\bigr)\bigr) \\ &= \bigl[\phi \bigl(x'(t) \bigr)x'(t) \bigr]^{T}_{0}- \int^{T}_{0}\phi\bigl(x'(t) \bigr)\,dx'(t)=0 \end{aligned}$$

and

3.8 $$ { } \int^{T}_{0}g\bigl(x(t-\tau)\bigr)x'(t)\,dt= \int^{T}_{0}g\bigl(x(t-\tau)\bigr)\,dx(t)= \int^{T}_{0}g\bigl(x(t-\tau )\bigr)\,dx(t-\tau)=0, $$

since $dx(t)=\frac{dx(t-\tau)}{d(t-\tau)}\,dt=dx(t-\tau)$.

Substituting (3.7) and (3.8) into (3.6), we have

3.9 $$ { } \int^{T}_{0}f\bigl(t,x(t)\bigr) \bigl\vert x'(t) \bigr\vert ^{2}\,dt= \int^{T}_{0}e(t)x'(t)\,dt. $$

From (3.9), we have

$$\biggl\vert \int^{T}_{0} f\bigl(t,x(t)\bigr) \bigl\vert x'(t) \bigr\vert ^{2}\,dt \biggr\vert = \biggl\vert \int^{T}_{0}e(t)x'(t)\,dt \biggr\vert . $$

From $(H_{4})$, we know

$$\biggl\vert \int^{T}_{0} f\bigl(t,x(t)\bigr) \bigl\vert x'(t) \bigr\vert ^{2}\,dt \biggr\vert = \int^{T}_{0} \bigl\vert f\bigl(t,x(t)\bigr) \bigr\vert \bigl\vert x'(t) \bigr\vert ^{2}\,dt\geq\gamma \int ^{T}_{0} \bigl\vert x'(t) \bigr\vert ^{2}\,dt. $$

Therefore, we can get

$$\begin{aligned} \gamma \int^{T}_{0} \bigl\vert x'(t) \bigr\vert ^{2}\,dt\leq{}& \int^{T}_{0} \bigl\vert e(t) \bigr\vert \bigl\vert x'(t) \bigr\vert \, dt \\ \leq{}& \biggl( \int^{T}_{0} \bigl\vert e(t) \bigr\vert ^{2}\,dt \biggr)^{\frac{1}{2}} \biggl( \int ^{T}_{0} \bigl\vert x'(t) \bigr\vert ^{2}\,dt \biggr)^{\frac{1}{2}} \\ ={}& \Vert e \Vert _{2} \biggl( \int^{T}_{0} \bigl\vert x'(t) \bigr\vert ^{2}\,dt \biggr)^{\frac{1}{2}}, \end{aligned}$$

where $\Vert e \Vert _{2}= (\int^{T}_{0} \vert e(t) \vert ^{2}\,dt )^{\frac{1}{2}}$. It is easy to see that there exists a positive constant $M_{1}'$ (independent of *λ*) such that

3.10 $$ { } \int^{T}_{0} \bigl\vert x'(t) \bigr\vert ^{2}\,dt\leq M_{1}'. $$

From (3.2) and (3.10), we have

3.11 $$\begin{aligned} { } x(t)&=x(t_{1})+ \int^{t}_{t_{1}}x'(s)\,ds\leq d_{2}+ \int^{T}_{0} \bigl\vert x'(t) \bigr\vert \,dt \\ &\leq d_{2}+\sqrt{T} \biggl( \int^{T}_{0} \bigl\vert x'(t) \bigr\vert ^{2}\,dt \biggr)^{\frac{1}{2}}\leq d_{2}+\sqrt{T} \bigl(M_{1}' \bigr)^{\frac{1}{2}}:=M_{1}. \end{aligned}$$

On the other hand, integrating both sides of (2.2) over $[0,T]$, we have

3.12 $$ { } \int^{T}_{0}\bigl[f\bigl(t,x(t)\bigr)x'(t)+g \bigl(x(t-\tau)\bigr)-e(t)\bigr]\,dt=0. $$

Therefore, from (3.10), (3.12) and $(H_{2})$, we have

3.13 $$\begin{aligned} &\int^{T}_{0} \bigl\vert g\bigl(x(t-\tau)\bigr) \bigr\vert \,dt \\ &\quad= \int_{g(x(t-\tau))\geq0}g\bigl(x(t-\tau)\bigr)\,dt- \int _{g(x(t-\tau))\leq0}g\bigl(x(t-\tau)\bigr)\,dt \\ &\quad =2 \int_{g(x(t-\tau))\geq0}g\bigl(x(t-\tau)\bigr)\,dt+ \int^{T}_{0}f\bigl(t,x(t)\bigr)x'(t)\,dt- \int ^{T}_{0}e(t)\,dt \\ &\quad \leq2 \int_{g(u(t-\tau))\geq0}\bigl(ax^{m}(t-\tau)+b\bigr)\,dt+ \int ^{T}_{0} \bigl\vert f\bigl(t,x(t)\bigr) \bigr\vert \bigl\vert x'(t) \bigr\vert \,dt+ \int^{T}_{0} \bigl\vert e(t) \bigr\vert \,dt \\ &\quad \leq2a \int^{T}_{0} \bigl\vert x(t-\tau) \bigr\vert ^{m}\,dt+2bT+ \biggl( \int^{T}_{0} \bigl\vert f\bigl(t,x(t)\bigr) \bigr\vert ^{2}\,dt \biggr)^{\frac{1}{2}} \biggl( \int^{T}_{0} \bigl\vert x'(t) \bigr\vert ^{2}\,dt \biggr)^{\frac{1}{2}} + \Vert e \Vert _{2}T^{\frac{1}{2}} \\ &\quad \leq2aM_{1}^{m}T+2bT+ \Vert f_{M_{1}} \Vert _{2} {M'}_{1}^{\frac{1}{2}}+ \Vert e \Vert _{2}T^{\frac{1}{2}}, \end{aligned}$$

where $f_{M_{1}}:=\max_{0\leq x(t)\leq M_{1}} \vert f(t,x) \vert , \Vert f_{M_{1}} \Vert _{2}:= (\int^{T}_{0} \vert f(t,x(t)) \vert ^{2}\,dt )^{\frac {1}{2}}$. As $x(0)=x(T)$, there exists a point $t_{2}\in[0,T]$ such that $x'(t_{2})=0$, while $\phi(0)=0$, from (3.10), (3.12) and (3.13), we have

3.14 $$\begin{aligned} \bigl\Vert \phi\bigl(x' \bigr) \bigr\Vert ={}&\max_{t\in[0,T]}\bigl\{ \bigl\vert \phi \bigl(x'(t)\bigr) \bigr\vert \bigr\} \\ ={}&\max_{t\in[t_{2},t_{2}+T]} \biggl\{ \biggl\vert \int^{t}_{t_{2}}\bigl(\phi\bigl(x'(s)\bigr) \bigr)'\,ds \biggr\vert \biggr\} \\ \leq{}& \int^{T}_{0} \bigl\vert f\bigl(t,x(t)\bigr) \bigr\vert \bigl\vert x'(t) \bigr\vert \,dt+ \int^{T}_{0} \bigl\vert g\bigl(x(t-\tau)\bigr) \bigr\vert \,dt+ \int ^{T}_{0} \bigl\vert e(t) \bigr\vert \,dt \\ \leq{}& \biggl( \int^{T}_{0} \bigl\vert f\bigl(t,x(t)\bigr) \bigr\vert ^{2}\,dt \biggr)^{\frac{1}{2}} \biggl( \int ^{T}_{0} \bigl\vert x'(t) \bigr\vert ^{2}\,dt \biggr)^{\frac{1}{2}}+ \int^{T}_{0} \bigl\vert g\bigl(u(t-\tau)\bigr) \bigr\vert \,dt \\ &{}+ \int ^{T}_{0} \bigl\vert e(t) \bigr\vert \,dt \\ \leq{}& 2 \bigl(aM_{1}^{m}T+bT+ \Vert f_{M_{1}} \Vert _{2} {M'}_{1}^{\frac{1}{2}}+ \Vert e \Vert _{2}T^{\frac{1}{2}} \bigr):={M'}_{2}. \end{aligned}$$

We claim that there exists a positive constant $M_{2}>M_{2}'+1$ such that, for all $t\in\mathbb{R}$,

3.15 $$ { } \bigl\Vert x' \bigr\Vert \leq M_{2}. $$

In fact, if $x'$ is not bounded, then from the definition of *α*, there exists a positive constant $M_{2}''$ such that $\alpha( \vert x' \vert )>M_{2}''$ for some $x'\in\mathbb{R}$. However, from $(A_{2})$, we have

$$\alpha\bigl( \bigl\vert x' \bigr\vert \bigr) \bigl\vert x' \bigr\vert \leq\phi\bigl(x'\bigr)x' \leq \bigl\vert \phi\bigl(x'\bigr) \bigr\vert \bigl\vert x' \bigr\vert \leq M_{2}' \bigl\vert x' \bigr\vert . $$

Then we can get

$$\alpha\bigl( \bigl\vert x' \bigr\vert \bigr)\leq M_{2}' \quad\mbox{for all } x\in\mathbb{R}, $$

which is a contradiction. So, (3.15) holds.

From (2.2), we have

3.16 $$ { } \bigl(\phi\bigl(x'(t+\tau)\bigr) \bigr)'+\lambda f\bigl(t+\tau,x(t+\tau)\bigr)x'(t+ \tau)+\lambda g\bigl(x(t)\bigr)=\lambda e(t+\tau). $$

Multiplying both sides of (3.16) by $x'(t)$ and integrating on $[\xi,t]$, here $\xi\in[0,T]$, we get

3.17 $$\begin{aligned} \lambda \int^{x(t)}_{x(\xi)}g(x)\,dx={}&\lambda \int^{t}_{\xi}g\bigl(x(s)\bigr)x'(s)\,ds \\ ={}&- \int^{t}_{\xi}\bigl(\phi\bigl(x'(s+\tau) \bigr)\bigr)'x'(s)\,ds-\lambda \int^{t}_{\xi}f\bigl(s+\tau ,x(s+\tau) \bigr)x'(s+\tau)x'(s)\,ds \\ &{}+\lambda \int^{t}_{\xi}e(s+\tau)x'(s)\,ds. \end{aligned}$$

By (3.14) and (3.15), we can get

$$\begin{aligned} &\biggl\vert \int^{t}_{\xi}\bigl(\phi\bigl(x'(t+\tau) \bigr)\bigr)'x'(s)\,ds \biggr\vert \\ &\quad \leq \int^{t}_{\xi } \bigl\vert \bigl(\phi \bigl(x'(s+\tau)\bigr)\bigr)' \bigr\vert \bigl\vert x'(s) \bigr\vert \,ds \\ &\quad\leq \bigl\Vert x' \bigr\Vert \int^{T}_{0} \bigl\vert \bigl(\phi \bigl(x'(t+\tau)\bigr)\bigr)' \bigr\vert \,dt \\ &\quad \leq\lambda \bigl\Vert x' \bigr\Vert \biggl( \int^{T}_{0} \bigl\vert f\bigl(t,x'(t) \bigr) \bigr\vert \,dt+ \int^{T}_{0} \bigl\vert g\bigl(x(t-\tau)\bigr) \bigr\vert \,dt+ \int ^{T}_{0} \bigl\vert e(t) \bigr\vert \,dt \biggr) \\ &\quad \leq 2\lambda M_{2}\bigl(aM_{1}^{m}T+bT+ \Vert f_{M_{1}} \Vert _{2}{M'}_{1}^{\frac{1}{2}}+ \Vert e \Vert _{2}T^{\frac{1}{2}}\bigr). \end{aligned}$$

Moreover, from (3.15), we have

$$\begin{aligned} & \biggl\vert \int^{t}_{\xi}f\bigl(s+\tau,x(s+\tau) \bigr)x'(t+\tau)x'(s)\,ds \Big|\leq \biggr\Vert x' \bigr\Vert ^{2}\sqrt{T} \biggl( \int^{T}_{0} \bigl\vert f\bigl(s+\tau,x(s+\tau) \bigr) \bigr\vert ^{2}\,ds \biggr)\\ &\phantom{\biggl\vert \int^{t}_{\xi}f\bigl(s+\tau,x(s+\tau) \bigr)x'(t+\tau)x'(s)\,ds \biggr\vert }\leq M_{2}^{2} \sqrt{T} \Vert f_{M_{1}} \Vert _{2}, \\ & \biggl\vert \int^{t}_{\xi}e(t+\tau)u'(t)\Big\vert \,dt \biggr\vert \leq M_{2}\sqrt{T} \Vert e \Vert _{2}. \end{aligned}$$

From (3.17), we have

3.18 $$\begin{aligned} { } \biggl\vert \int^{u(t)}_{u(\xi)}g_{0}(u)\,du \biggr\vert &\leq M_{2}\bigl(2aM_{1}^{m}T+2bT+2 \Vert f_{M_{1}} \Vert _{2}{M'}_{1}^{\frac{1}{2}}+M_{2} \sqrt{T} \Vert f_{M_{1}} \Vert _{2} +3\sqrt{T} \Vert e \Vert _{2}\bigr) \\ &:=M'_{3}. \end{aligned}$$

From $(H_{3})$, we know that there exists a constant $M_{3}>0$ such that

3.19 $$ { } x(t)\geq M_{3}, \quad \forall t\in[\xi,T]. $$

Similarly, we can consider $t\in[0,\xi]$.

Let $E_{1}<\min\{d_{1},M_{3}\}$, $E_{2}>\max\{d_{2}, M_{1}\}$, $E_{3}>M_{2}$ be constants, from (3.11), (3.15) and (3.19), we can get that the periodic solution *x* to (2.2) satisfies

$$ E_{1}< x(t)< E_{2}, \qquad\bigl\Vert x' \bigr\Vert < E_{3}. $$

Then condition (1) of Lemma 2.1 is satisfied. For a possible solution *C* to equation

$$g(C)-\frac{1}{T} \int^{T}_{0}e(t)\,dt=0, $$

it satisfies $E_{1}< C<E_{2}$. Therefore, condition (2) of Lemma 2.2 holds. Finally, we consider condition (3) of Lemma 2.2 is also satisfied. In fact, from $(H_{1})$, we have

$$g(E_{1})-\frac{1}{T} \int^{T}_{0}e(t)\,dt>0 $$

and

$$g(E_{2})-\frac{1}{T} \int^{T}_{0}e(t)\,dt< 0. $$

So condition (3) is also satisfied. By application of Lemma 2.2, we get that (1.4) has at least one positive periodic solution. □

## Main results (II): periodic solution of (1.4) with repulsive singularity

In this section, we consider (1.4) in the case that $f(t,x)\equiv f(x)$. Then (1.4) can be written as

4.1 $$ { } \bigl(\phi\bigl(x'(t)\bigr)\bigr)'+f \bigl(x(t)\bigr)x'(t)+g\bigl(x(t-\tau)\bigr)=e(t). $$

We will discuss the existence of a positive periodic solution for (4.1) with repulsive singularity.

### Theorem 4.1

*Assume that conditions* $(A_{1})$ *and* $(A_{2})$ *hold*. *Suppose that the following conditions hold*:

$(H_{1}^{*})$
*There exist constants* $0< d_{1}^{*}< d_{2}^{*}$ *such that* $g(x)<0$ *for* $x\in(0,d_{1}^{*})$ *and* $g(x)>0$ *for* $x\in(d_{2}^{*},+\infty)$.
$(H_{2}^{*})$
$\int^{T}_{0}e(t)\,dt=0$.
$(H_{3}^{*})$
(*Repulsive singularity*) $\lim_{x\to 0^{+}}\int^{1}_{x}g(s)\,ds=-\infty$.
$(H_{4}^{*})$
*There exists a constant* $\gamma^{*}>0$ *such that* $\inf_{x\in\mathbb{R}} \vert f(x) \vert \geq\gamma^{*}>0$.
*Then* (4.1) *has at least one positive* *T*-*periodic solution*.

### Proof

Firstly, we embed equation (4.1) into the following equation family with a parameter $\lambda\in(0,1]$:

4.2 $$ { } \bigl(\phi\bigl(x'(t)\bigr)\bigr)'+ \lambda f\bigl(x(t)\bigr)x'(t)+\lambda g\bigl(x(t-\tau)\bigr)=\lambda e(t). $$

Integrating both sides of (4.2) over $[0,T]$, from $(H_{2}^{*})$, we have

4.3 $$ { } \int^{T}_{0}g\bigl(x(t-\tau)\bigr)\,dt=0. $$

From the continuity of *g*, we know there exists $t_{1}^{*}\in[0,T]$ such that

$$g\bigl(x\bigl(t_{1}^{*}-\tau\bigr)\bigr)=0. $$

Let $t_{3}=t_{1}^{*}-\tau$, from assumption $(H_{1})$ we can get

$$d_{1}^{*}\leq x(t_{3})\leq d_{2}^{*}. $$

We follow the same strategy and notation as in the proof of Theorem 3.1. We know that there exists $M_{1}^{*}>0$ such that

$$x(t)\leq M_{1}^{*}. $$

Next, we prove that there exists a positive constant $M_{2}^{*}$ such that $\Vert x' \Vert \leq M_{2}^{*}$.

In fact, we get from (4.3) that

4.4 $$\begin{aligned} { } \int^{T}_{0} \bigl\vert g\bigl(x(t-\tau)\bigr) \bigr\vert \,dt={}& \int_{g(x(t-\tau))\geq0}g\bigl(x(t-\tau)\bigr)\,dt- \int _{g(x(t-\tau))\leq0}g\bigl(x(t-\tau)\bigr)\,dt \\ ={}&2 \int_{g(x(t-\tau))\geq0}g\bigl(x(t-\tau)\bigr)\,dt \\ \leq{}&2 \int^{T}_{0}g^{+}\bigl(x(t-\tau)\bigr)\,dt, \end{aligned}$$

where $g^{+}(x)=\max\{g(x),0\}$. Since $g^{+}(x(t-\tau))\geq0$, from $(H_{1}^{*})$ we know $x(t-\tau)\geq d_{2}^{*}$. Then we have

4.5 $$\begin{aligned} { } \int^{T}_{0} \bigl\vert g\bigl(x(t-\tau)\bigr) \bigr\vert \,dt\leq{}&2 \int^{T}_{0}g^{+}\bigl(x(t-\tau)\bigr)\,dt \\ \leq{}&2T \bigl\Vert g^{+}_{M_{1}} \bigr\Vert , \end{aligned}$$

where $\Vert g^{+}_{M_{1}} \Vert =\max_{d_{2}^{*}\leq x\leq M_{1}}g^{+}(x)$.

As $x(0)=x(T)$, there exists a point $t_{4}\in[0,T]$ such that $x'(t_{4})=0$, which $\phi(0)=0$, we have

4.6 $$\begin{aligned} { } \bigl\vert \phi\bigl(x'(t) \bigr) \bigr\vert ={}& \biggl\vert \int^{t}_{t_{4}}\bigl(\phi\bigl(x'(s)\bigr) \bigr)'\,ds \biggr\vert \\ \leq{}&\lambda \biggl( \int^{T}_{0} \bigl\vert f\bigl(x(t)\bigr) \bigr\vert \bigl\vert x'(t) \bigr\vert \,dt+ \int^{T}_{0} \bigl\vert g\bigl(x(t-\sigma )\bigr) \bigr\vert \,dt+ \int^{T}_{0} \bigl\vert e(t) \bigr\vert \,dt \biggr) \\ \leq{}&\lambda \biggl( \Vert f_{M_{1}} \Vert T^{\frac{1}{2}} \biggl( \int ^{T}_{0} \bigl\vert x'(t) \bigr\vert ^{2}\,dt \biggr)^{\frac{1}{2}}+2T \bigl\Vert g^{+}_{M_{1}} \bigr\Vert +T^{\frac{1}{2}} \Vert e \Vert _{2} \biggr) \\ \leq{}&\lambda \bigl( \Vert f_{M_{1}} \Vert T^{\frac{1}{2}} \bigl(M_{1}' \bigr)^{\frac {1}{2}}+2T \bigl\Vert g^{+}_{M_{1}} \bigr\Vert +T^{\frac{1}{2}} \Vert e \Vert _{2} \bigr). \end{aligned}$$

Thus, from (3.15) we know that there exists some positive constant $M_{2}^{*}$ such that

4.7 $$ { } \bigl\Vert x' \bigr\Vert \leq M_{2}^{*}. $$

The proof left is the same as that of Theorem 3.1. □

## Examples

### Example 5.1

Consider the following *ϕ*-Laplacian Liénard equation with attractive singularity

5.1 $$ { } \bigl(\phi\bigl(x'(t)\bigr)\bigr)'+ \bigl(5x^{6}\cos^{2}t+10\bigr)x'(t)-3x^{m}(t- \tau)+\frac{1}{x^{\kappa}(t-\tau)}=e^{\cos^{2}t}, $$

where $\phi(u)=ue^{ \vert u \vert ^{2}}$, *τ* is a positive constant and $0<\tau <T$, $m\geq0$ and $\kappa\geq1$.

Comparing (5.1) to (1.4), it is easy to see that $g(x)=-3x^{m}(t-\tau)+\frac{1}{x^{\kappa}(t-\tau)}$, $f(t,x)=5x^{6}\cos^{t}+10$, $e(t)=e^{\cos^{2}t}, T=\pi$. Obviously, we get

$$\bigl( xe^{ \vert x \vert ^{2}}-ye^{ \vert y \vert ^{2}}\bigr) (x-y)\geq\bigl( \vert x \vert e^{ \vert x \vert ^{2}}- \vert y \vert e^{ \vert y \vert ^{2}}\bigr) \bigl( \vert y \vert - \vert y \vert \bigr)\geq0 $$

and

$$\phi(x)\cdot x= \vert x \vert ^{2}e^{ \vert x \vert ^{2}}. $$

So, conditions $(A_{1})$ and $(A_{2})$ hold. Moreover, it is easy to see that there exist constants $d_{1}=0.1$ and $d_{2}=1$ such that $(H_{1})$ holds. $g(x)\leq3x^{m}+1$, here $a=3, b=1$, condition $(H_{2})$ holds. Consider $\vert f(t,x) \vert = \vert 5x^{6} \cos^{2}t+10 \vert \geq10:=\gamma$, so, condition $(H_{4})$ is satisfied. Next, we consider that condition $(H_{3})$ is also satisfied. In fact, $\lim_{x\to 0^{+}}\int^{1}_{x}g(s)\,ds=\lim_{x\to 0^{+}}\int^{1}_{x}(-x^{m}+\frac{1}{x^{\kappa}})\,ds=+\infty$, thus, condition $(H_{3})$ holds. Therefore, by Theorem 3.1, we know that (5.1) has at least one positive *π*-periodic solution.

### Example 5.2

Consider the *ϕ*-Laplacian Liénard equation with repulsive singularity:

5.2 $$ { } \bigl(\phi\bigl(x'(t)\bigr)\bigr)'+ \bigl(x^{4}+3\bigr)x'(t)+5x^{m}(t-\tau)- \frac{1}{x^{\kappa}(t-\tau)}=\sin t, $$

where relativistic operator $\phi(u)=\frac{u}{\sqrt{1- (\frac{ \vert u \vert }{c} )^{2}}}$, here *c* is the speed of light in the vacuum and $c>0$, *τ* is a constant and $0\leq\tau< T$, $m\geq0$.

It is clear that $T=2\pi$, $g(x)=5x^{m}(t-\tau)-\frac{1}{x^{\kappa}(t-\tau)}$, $f(x)=x^{4}+3$, $e(t)=\sin t$. It is obvious that $(H_{1}^{*})$-$(H_{4}^{*})$ hold. Now we consider conditions $(A_{1})$ and $(A_{2})$.

$$\biggl(\frac{u}{\sqrt{1- (\frac{ \vert u \vert }{c} )^{2}}}-\frac{v}{\sqrt {1- (\frac{ \vert v \vert }{c} )^{2}}} \biggr) (u-v)\geq 0 $$

and

$$\phi(u)\cdot u=\frac{ \vert u \vert ^{2}}{\sqrt{1- (\frac{ \vert u \vert }{c} )^{2}}}. $$

Then conditions $(A_{1})$ and $(A_{2})$ hold. Therefore, by Theorem 4.1, we know that (5.2) has at least one positive periodic solution.
